# Response—A Critical Response to “Discourse Communities and the Discourse of Experience”

**DOI:** 10.1007/s11673-021-10156-6

**Published:** 2022-04-01

**Authors:** Paul Macneill

**Affiliations:** Sydney Health Ethics, University of Sydney, Sydney, NSW 2006 Australia

**Keywords:** Dialogue, Discourse community, Ethical community, Bakhtin, Art, Language, Ethics

## Abstract

In their article Little, Jordens, and Sayers developed the notion of “discourse communities”—as groups of people who share an ideology and common “language”—with the support of seminal ideas from M.M. Bakhtin. Such communities provide benefits although they may also impose constraints. An ethical community would open to others’ discourse and be committed to critique. Those commitments may counter the limitations of discourse communities. Since their paper was published in 2003, the notion of “discourse communities” has been widely adopted and applied in healthcare and beyond. Their ideas were influential in the founding of an ethics centre in Sydney and contributed to articulating the values which underpin this journal. This commentary notes that an ethical community is fragile in responding to current onslaughts on truth and meaning—potencies inherent in discourse communities. The essay takes Bakhtin’s ideas further to explore intrinsic forces at play in dialogue, language, and art. This leads to discussing the centrality of ethics in Bakhtin’s thought. For him, the essence of discourse is a dialogic exchange which comprises both art and ethics. It is *art* in that *self* and *other* are created in the exchange. It is *ethical* in that “I” am *answerable* to the other, as a phenomenological reality, in the moment of intersubjectivity.

## Introduction

This Critical Response to “Discourse communities and the discourse of experience” (Little, Jordens, and Sayers 2003) is presented in the following sections:

A. Travelling with the authors in exploring discourse communities;
B. Exploring the nature of those communities, as the authors conceive of them, in terms of both the needs and purposes they serve and their constraints on members;
C. Considering the extent to which discourse communities may have served the needs and ideals of the authors themselves—which leads into discussing “the possibility of an ethical community”—and what would be required of such a community;
D. Discussing the ideologies and values (and fragility) of discourse communities in responding to a current onslaught on truth and meaning; and
E. Returning to the Russian theorist—M.M. Bakhtin—from whom the authors drew seminal ideas in support of their approach to discourse communities.

The commentary concludes by proposing a further journey—to the interior of the terrain that Bakhtin explored—by drawing on his view of ethics and aesthetics in the moment of intersubjectivity.

### **A. Discourse Communities**

In the “Discourse communities” paper, the authors developed a notion of *discourse communities* as groups of people who share an ideology that is expressed in a common vocabulary of words with meanings that are understood in the same way by members of that group. The examples they give include the medical profession whose members “share common concepts of disease-causation and disease treatment” (74) along with other professions and members of trade groups such as plumbers and electricians. Discourse communities also include less clearly defined groups such as racists or postmodernists; and followers of a particular religion, members of church groups, family groups, and those with sporting affiliations. Membership within each community is “defined by a particular use of language” (74). Understood in this way, it is apparent that most of us belong to *many* discourse communities: as family members, members of professional or trade groups, religious followers (or resolute atheists), and *aficionados* of one or more sporting codes.

The authors observe that each of these communities “draw on the same linguistic resources, the same lexicon and grammar, but construe meanings in different ways” (80). Yet we move between these communities easily and understand the same words as having different meanings within the context of each discourse community. A word—such as “shock”—will be understood among family members and friends differently from the way it is understood by doctors, seismologists, or electricians.

There is also a suggestion in this paper, that membership of different discourse communities may shape the way in which we experience events in our lives or at least the way in which we describe those experiences, although they step carefully around the “epistemological status of experience” whilst acknowledging experience is important as “the matrix upon which meaning and values are mounted” (77, 78).^1^ The authors remark that “extreme experience,” such as torture, natural disasters, or suffering cancer, can alter one’s thinking, perceptions, and memories and be accompanied by an immediacy of intense emotions “that cannot be freely communicated to others who have not had similar experiences” (76). Extreme experience “challenges our sense of identity in all its elements” (76) leaving us vulnerable and exposed.

The paper emerged from the authors’ work with cancer survivors and their carers. They observed different discourse communities in that context and differences in the discourse of those communities in speaking of cancer. Cancer patients and their lay-carers spoke of their experience as “victims of circumstance, people to whom things happen” (78) and as being caught in systems “over which they have little control” (78). By contrast Little et al. found that “Administrators, bureaucrats, business people, lawyers and insurance representatives” spoke “as agents of change to the systems in which they work” and of how “they made events into opportunities” (77-78). Doctors and other healthcare workers however, “tell stories which sit somewhere betwixt and between” (78). Whilst they are “clearly agents of change for their patients” and are “involved morally with the extreme experiences of their patients”, they are also “profoundly affected by the vagaries and demands of the administrative and economic systems within which they must work” (78). The proposition that emerges from this analysis is that the “basic division between discourse communities has to be recognized and understood before there can be any prospect of real dialogue between the representatives of different interests in health care” (79).

Since this paper was published, the idea of discourse communities has been adopted widely in the literature. There have been numerous citations.^2^ Many of them relate directly to its basic proposition—the need for recognition of differences between different discourse communities within healthcare as a means for promoting dialogue between them (Christine, 2016; Kvarnström and Cedersund, 2006; Torjuul, 2009). The notion has been applied to indigenous health in a study of cultural differences between health professionals providing cancer care to Aboriginal people (Newman et al. 2013).

Beyond health, the “Discourse communities” paper has been referred to across a wide variety of topics. These include the “troubles” in Ireland (McAuley and Tonge 2011; Tonge et al. 2013); alcohol use among college students in the mid-West of the United States (Russell and Arthur 2016); masculinity, crime, and culture in Australia (Seidler 2010); and theology: understanding the reintegration of marginalized widows in the Old Testament (Ruth and Naomi) in terms of their acceptance into discourse communities (Matthews 2006). It is readily apparent that the notion of discourse communities has broad appeal and application. It is an idea that—having been disseminated—appears blindingly obvious. Yet it was not (apparently) so obvious back then, or had not been so clearly articulated, prior to its publication by Little, Jordens, and Sayers in 2003.

### **B. Discourse Communities: Needs and Purposes and Concomitant Constraints**

Discourse communities fulfil a basic need in that, as “social and societal animals,” most of us “feel the need to be members of communities” and belong to groups “that ‘speak the same language’. There is comfort in belonging” (74, 80). However, there are also risks. As they put it, “Membership … potentially constrains what we should think” (74) or at least constrains what we say we think. The authors refer to this as a colonizing process in which we are obliged to understand and speak in words with fixed meanings and limited usages. In some cases, “discourse communities … can easily slip from benign intent into exploitation” (82) leading vulnerable people, such as those discussed above, to a sense of being “victims of circumstance” (73). This is also reported by patients with Type 2 diabetes (Parry et al. 2006); and by injured, unwell, and disabled employees in an international IT company (Allender, Colquhoun, and Kelly 2006).

The authors note that the communicative problems between different discourse communities in health are sustained by a “demotic, centrifugal force” which supports difference and keeps things apart. This is a reference to the work of Russian literary critic M.M. Bakhtin. The authors also refer to a counter force as “a multiplicity of social voices and a wide variety of their links and interrelationships”—again a reference to Bakhtin (Bakhtin and Holquist 1981 263). These include “healing narratives which may restore autonomy to the disempowered” (Little, Jordens, and Sayers 2003, 75). Although the authors draw on Bakhtin’s ideas, I have left a more detailed treatment of his ideas until the last two sections of this critical response because I want to place Bakhtin in the context of *his* discourse, rather than confine his ideas to supporting the notion of discourse communities, as the authors have done—as was appropriate to their purpose.^3^

My point here is that there are other forces at work which counter the pull toward identifying solely with a particular community. We could refer to them as “centripetal forces that strive to make things cohere” (Bakhtin and Holquist 1981, xviii). A “centripetal force” moves toward a centre (OED), which is an apt description of the force prompting these authors to explore the possibility of an ethical community.

### **C. The Possibility of an Ethical Community**

The biographical notes which accompany this paper describe Emma-Jane Sayers as a cancer survivor. She has been on the executive of “an organization that provides support services for young adults diagnosed with cancer. She represents cancer survivors on a number of national organizations in Australia” (86). One might assume that Emma-Jane had opportunities to share her experience of cancer with others who had similar experiences—an opportunity which (in the authors’ words) “can be liberating, and even therapeutic” (83). This is an example of a benefit that derives from belonging to a discourse community.

Emma-Jane was also uniquely placed to gain from speaking across discourse communities in that her work (with Miles Little and Christopher Jordens) included analysing interviews with cancer patients, clinical carers, and health policymakers. One benefit, from this broader view, is the “possibility of creative dialogue.” One can imagine that participating in their research provided Emma-Jane with the opportunity to understand what “care” meant within those different discourse communities, an understanding not easily gained by fellow members of a cancer survivor discourse community.

Professor Little, on retiring as a practising surgeon, set up the Centre for Values, Ethics and the Law in Medicine (VELiM) at the University of Sydney in 1996, as its Director (until 2003). It is apparent that the “Discourse Communities” paper is an expression of his deep commitment to open discourse. He had previously published *Humane Medicine*, a book which argued for a shift in emphasis from biomedicine to humane medicine (Little 1995). The values that are important to him were captured in the name of the Centre—with particular emphasis on *values* and *ethics*. Christopher Jordens joined the Centre in 1997, initially as a researcher on the various studies referred to in the “Discourse” paper. From 2006 he played a key role in setting up and running the postgraduate coursework programme in bioethics including major units of study. He was committed to the values of the Centre and particularly to the practice of conversation as “openness to one’s interlocutor: an openness that entails a risk: a risk that you’ll be changed by what they say” (Jordens 2021). Although their paper focusses on *discourse*, that can be understood within a wider commitment to *open conversation*. Chris Jordens, in a recent tribute to Miles Little, described him as “a practitioner of conversation.” This found expression in the Centre “in its research; in its teaching; in its consultation and public engagement and, more generally, in its collegial culture” (ibid). This has been my experience of the Centre also: conversation among colleagues sharing a common interest, conversation of the best kind—about important issues in medicine and healthcare—skilfully facilitated by Miles who invited and encouraged many perspectives within a supportive atmosphere.

These brief glimpses from the authors’ biographies provide some background and understanding of their commitment to the values expressed in the paper, most clearly in the section “The possibility of an ethical community.” An ethical community is presented as a “species of discourse community” which interrogates ideologies including its own. As an interdisciplinary community it would “not be committed to any one model of ethics” but would be committed to “processes of ethical examination using many models.” Its members would be open-minded and dedicated to examining “underlying values that sustain and justify ethical endeavour of all kinds.” It would also be practical in testing “its conclusions by political and social action … in the light of actual happenings and interactions” (82).

It is a remarkable achievement that VELiM—recently renamed “Sydney Health Ethics” (SHE)—has been sustained as an ethical community (in just this way) for twenty-five years. All things change however. Recent events threaten to undermine the community as a discrete entity. SHE has lost its formal status as a Centre, and its staff members have been relocated from a separate building and combined with staff of the School of Public Health. Whether or not it survives as an entity, its manifestation and its maintenance for twenty-five years has “spread … its sphere of influence” (Little, Jordens, and Sayers 2003, 83). As Chris expressed this idea, it is an accomplishment that has created “the possibility of an enduring centre that is constituted if not through institutional recognition, then through (among other things) the practice of genuine conversation” (Jordens op. cit.).

### **D. Ethical Community in Relation to a Current Issue of Truth and Meaning**

In the spirit of their paper, I want to test the “possibility of an ethical community” in responding to a current onslaught on truth and meaning. Mark Danner (2021) recently wrote of “Trump’s Big Lie that the election was stolen” and the subsequent ransacking of the Capitol Hill building. Yet “we have thus far ignored the truth.” The evidence of Trump’s complicity “did not persuade most of his supporters to abandon their overwhelmingly partisan version of events” (Section 1, ¶8 and 9). This is an example of the dark side of discourse communities, with dire consequences. “[T]here is no shared reality” about Capitol Hill. “Nor is there a shared reality about the integrity of the election or of the legitimacy of the president it produced. To millions of Americans the legitimate president remains Donald Trump” (Danner 2021, Section 1, last para). This raises a profound question: Is there any basis for determining truth or facts upon which we may agree—beyond partisanship?

I have been considering that question in relation to the “the possibility of an ethical community.” Little, Jordens, and Sayers describe an ethical community as having “no special intellectual domain in which it operates. It would hold equal engagement with science, aesthetics, the spiritual, the human sciences and philosophy” (82-83). Whilst I applaud that as an ideal, it offers no firm foundation for determining a shared reality or even a basis for finding agreement over historical “facts” such as the holocaust or the ransacking of Capitol Hill. Admittedly, this is to raise fraught issues of epistemology and ontology going back to the beginnings of Greek philosophy. It is an issue that is well captured in the debate between Gadamer and Habermas last century (Mendelson 1979). I side with Habermas and accept that we are capable, through reflecting on our own prejudices (embedded as we are in an historical, cultural, and social position), of lessening their influence (Daniels 2020). As I read their paper, Little, Jordens, and Sayers also put their faith in this kind of reflection. Clearly (as they state) there are prerequisites: an open mind; a commitment to continual, reflexive critique; and a willingness to be seek out and replace fault and weakness. Not all of us possess those dispositions. Consequently, an ethical community is fragile, as is democracy in the face of the marauding herds. In a similar vein, the authors note that “Habermas has for many years examined the possible interface between discourse and ethics, and he knows well the difficulties of maintaining the force of critique in the interactions of politics” (83). Yet it is important to recognize these fragilities in order to bolster and maintain the virtues of openness and critique.

### **E. Discourse in the Novel: M.M. Bakhtin**

The authors draw on M.M. Bakhtin for concepts relating to language, to substantiate their approach to discourse communities. Mikhail Mikhailovich Bakhtin (1895—1975) was a Russian philosopher, literary critic, and linguist who wrote voluminously, although much of his writing has been lost. That which has survived has garnered a late following among linguists and literary theorists beyond Russia. The lost material, and publication of his remaining texts, out of their chronological sequence, has however, “led to a partial view of Bakhtin, which obscured the centrality of ethics in his thought” (Çalişkan 2006, 2).

Little et al. quote from Bakhtin’s essay “Discourse in the novel” which was published in English as one of four essays in *The Dialogic Imagination* (Bakhtin and Holquist 1981). They take the concept *monoglossia* from this essay to mean the particular language and expression that evolves within a single discourse community, and they contrast this with *heteroglossia* which recognizes discourse across many different communities.^4^ In “Discourse in the novel” however, Bakhtin used the term *heteroglossia* as a characteristic feature of novels. Bakhtin was a critic of all previous attempts to systematize the novel as a genre—and drew attention to what is *novel* about novels: they break the conventions that traditional scholars had previously tried to impose on the novel (ibid 263). *Discourse* in the novel is *heteroglossic* in that the “novel orchestrates … the speech of characters” and many other elements and permits them to “enter the novel.” For Bakhtin, the “fundamental condition, that which makes a novel a novel … is the *speaking person and his discourse*” (ibid, 332, italics in the original). This critique of literary analysis also extends to language itself. “Every concrete utterance of a speaking subject” contains a tension between conforming with normative standards of language (*monoglossia*) whilst also participating in “speech diversity” (*heteroglossia*) in that it is individualized (ibid, 272). Subjected to these dynamic forces, language is constantly evolving and breaking from the moulds of conventional analysis. It refuses to be confined by rules (of meaning, style, or grammar). This critique is equally applicable to ethics.

## Discussion and Conclusion

In the Introduction I proposed a further journey—to the interior of the terrain that Bakhtin explored—by drawing on his view of ethics and aesthetics in the moment of intersubjectivity.

For Bakhtin, *heteroglossia* has a primary sense as a feature of any communicative exchange between two (or more) people. The word is a translation of the Russian *raznorečie*—from *raznyĭ* (meaning “other,” “separate,” “different,” or “various”) and *reč* (meaning “word” or “speech”). Any “speech act” or “utterance” is understood by Bakhtin as one side of a dialogue with an *other* “I.” If one is to relate to another “I” as a subject, not just as an object, then there is an “aesthetic invention” or act of creativity “that occurs between subjects in actual dialogues” (Nielsen 1995, 807). Moreover, this is also an ethical exchange in the sense that one is answerable to the other. This is based on the phenomenological experience of dialogue with another. I speak in anticipation of the other’s response, and likewise that *other* anticipates mine. Without that “answerability” there is no genuine engagement. For Bakhtin, dialogue is both ethical and aesthetic. It is ethical in that one is answerable for one’s utterances and actions in relation to another (Bøe et al. 2013). And it is aesthetic in that the exchange is also an act of *self* and *other* creativity. More precisely, the dialogue is “trans-subjective” in the sense that both *self* and *other* are subjects (ibid). Yet both are also objects to each other. My image of you, and yours of me, are influential and creative in the exchange.

The implications of Bakhtin’s view of self, art, and answerability, are radical.^5^ In one of his earlier works, Bakhtin had asked, “what guarantees the inner connection of the constituent elements of a person? Only the unity of answerability” (Bakhtin 1990, 1). His conception is also radical in that *self* is ever in flux: “I occupy a place in once-occurrent Being that is unique and never-repeatable, a place that cannot be taken by anyone else and is impenetrable for anyone else” (Bakhtin 1993, 40). These ideas are consistent with his critique of all attempts to systematize the novel as a genre and to systematize language itself. There are opposing and dynamic forces at work in the very instant of discourse, and these forces break the containment of any system. He is similarly critical of grand systems in ethics. There could be no authentic ethics founded on morality external to oneself (such as Kant’s categorical imperative) because it would require *dying* as an individual. For Bakhtin “ethics must start from the unique and once-occurent human deed—my own individually” (Çalişkan 2006, 4).

I have previously argued for balancing normative ethics by sensing the aesthetic and by understanding ethics as art (Macneill 2014, 2017, and 2020). Bakhin takes such notions much further. Ethics *is* art in a profound sense, in that one’s *self* and *others* are created (and re-created) in dialogical exchanges. What makes that art profoundly *ethical* is that “I” am answerable to *others* in those exchanges, and continue to be answerable across a flux of time. In this sense, Bakhtin’s ideas are revolutionary. In putting them forward, I am emboldened by Little, Jordens, and Sayers’ open-minded commitment to examining many models of ethics. The influence of these authors and the values of the community they founded—along with the values of the Otago Bioethics Centre—are evident in the commitment of the *Journal of Bioethical Inquiry* to ethics as “dialogue” and to promoting understanding by listening to “diverse voices in a global conversation [across] geographical borders” (*Journal of Bioethical Inquiry*^2021^). Given those values, Bakhtin’s ethical understandings are worthy of examination by the journal’s wide community of readers.

## Footnotes

1 To express that idea through the words of one commentator: experience is “a template upon which people’s personal values and subjective feelings are based” (Samanhudi 2020, 50).

2 From a search, across both ResearchGate and Google Scholar, I substantiated 54 references comprising 33 journal articles (not including those by the authors and associates), 10 books, and 11 theses (including PhD and Masters dissertations) from across widely dispersed geographical areas.

3 Little et al. are not the first authors to appropriate Bakhtin to their purpose. His ideas are “malleable” which is “part of the reason” that his “concepts can be revised, appropriated, and adapted” for other purposes (Cobb 2019, 42).

4 Monoglossia derives from “mono” (single) and “glossia” (tongue, speech, or language). Heteroglossia derives from hetero (“other”) and means *other* language or speech. Bakhtin however, uses the term *heteroglossia* in a number of different senses.

5 In the space available, I am unable to more fully develop those implications, although I am doing so in a paper in preparation.
